# Microarray profiling for differential gene expression in PMSG-hCG stimulated preovulatory ovarian follicles of Chinese Taihu and Large White sows

**DOI:** 10.1186/1471-2164-12-111

**Published:** 2011-02-16

**Authors:** Xiaojie Sun, Shuqi Mei, Hu Tao, Guodong Wang, Lina Su, Siwen Jiang, Changyan Deng, Yuanzhu Xiong, Fenge Li

**Affiliations:** 1 Key Laboratory of Pig Genetics and Breeding of Ministry of Agriculture & Key Laboratory of Agricultural Animal Genetics, Breeding and Reproduction of Ministry of Education, Huazhong Agricultural University, Wuhan 430070, PR China; 2 Hubei Key Laboratory of Animal Embryo Engineering and Molecular Breeding, Hubei Academy of Agriculture Science, Wuhan, 430064, PR China

## Abstract

**Background:**

The Chinese Taihu is one of the most prolific pig breeds in the world, which farrows at least five more piglets per litter than Western pig breeds partly due to a greater ovulation rate. Variation of ovulation rate maybe associated with the differences in the transcriptome of Chinese Taihu and Large White ovaries. In order to understand the molecular basis of the greater ovulation rate of Chinese Taihu sows, expression profiling experiments were conducted to identify differentially expressed genes in ovarian follicles at the preovulatory stage of a PMSG-hCG stimulated estrous cycle from 3 Chinese Taihu and 3 Large White cycling sows by using the Affymetrix Porcine Genechip™.

**Results:**

One hundred and thirty-three differentially expressed genes were identified between Chinese Taihu and Large White sows by using Affymetrix porcine GeneChip (*p *≤ 0.05, Fold change ≥ 2 or ≤ 0.5). Gene Ontology (GO) analysis revealed that these genes belonged to the class of genes that participated in regulation of cellular process, regulation of biological process, biological regulation, developmental process, cell communication and signal transduction and so on. Significant differential expression of 6 genes including *WNT10B *and *DKK2 *in the WNT signaling pathway was detected. Real-time RT-PCR confirmed the expression pattern in seven of eight selected genes. A search of chromosomal location revealed that 92 differentially expressed transcripts located to the intervals of quantitative trait loci (QTLs) for reproduction traits. Furthermore, SNPs of two differentially expressed genes- *BAX *and *BMPR1B *were showed to be associated with litter size traits in Large White pigs and Chinese DIV line pigs (*p *≤ 0.1 or *p *≤ 0.05).

**Conclusions:**

Our study detected many genes that showed differential expression between ovary follicles of two divergent breeds of pigs. Genes involved with regulation of cellular process, regulation of biological process, in addition to several genes not previously associated with ovarian physiology or with unknown function, were differentially expressed between two breeds. The suggestive or significant associations of *BAX *and *BMPR1B *gene with litter size indicated these genetic markers had the potentials to be used in pig industry after further validation of their genetic effects. Taken together, this study reveals many potential avenues of investigation for seeking new insights into ovarian physiology and the genetic control of reproduction.

## Background

Reproductive traits are of primary interest in livestock because they play a major role in efficiency of production. Selection for increased number of offspring has been employed in pigs with only limited success because of its low heritability and sex-limited nature [1]. Genetic characterization of litter size and its components (e.g. ovulation rate and embryo survival) will increase our understanding of the underlying physiology and could enhance genetic improvement through use of marker-assisted selection (MAS) [2]. In the past several decades, the reproductive strategy of the Chinese Taihu pigs, a breed which farrows three to five more piglets per litter than American or European pig breeds, has come under intense scrutiny [3,4]. The greater litter size at farrowing in multiparous Chinese Taihu sows is due, in part, to a greater ovulation rate, a greater embryonic survival, a lower fertilization failure rate, and a greater uterine capacity [5-7].

In order to isolate the factors controlling the component traits of litter size, the differentially expressed (DE) genes were characterized during conceptus transformation, in Meishan-Landrace conceptuses and endometrial tissue when compared with conventional Landrace sows, in the porcine endometrium between pregnant and non-pregnant sows, and in the Erhualian and Large White placenta [8-11]. Furthermore, DE genes were identified in porcine ovarian follicles of multiparous sows on 12 d to 14 d of the estrous cycle between a line of pigs selected for an index of ovulation rate and embryo survival and its randomly selected control line [2,12]. However, the DE genes in Chinese Taihu and Large White preovulatory follicles remain unexplored.

To develop a broader view of the gene expression in preovulatory ovary and to identify the key genes involved in ovulation, we used Affymetrix microarrays to screen the genes differentially expressed in preovulatory follicles from Chinese Taihu and Large White sows simulated by hCG at 80 h after PMSG administration. Bioinformatics analysis has revealed these DE genes were involved in important biological processes such as reproduction and the DE genes were then *in silico *mapped to quantitative trait loci (QTL) regions related to reproduction traits. Real-time RT-PCR was used to confirm the expression profiles of various genes. And association analyses of two DE genes (*BAX *and *BMPR1B *gene) with litter size were carried out to screen the molecular markers for litter size. This research identified candidate genes, molecular markers and pathways associated with ovulation rate, and gained a further insight into the genetic basis of the prolificacy of Chinese Taihu pigs.

## Results

### Transcriptome analysis

Expression profiling experiments of Large White and Chinese Taihu ovary follicles were conducted by a commercial Affymetrix Porcine Genechip including 24,123 probe sets, which represent 23,999 transcripts and 124 controls. The transcriptome of ovarian follicles from Chinese Taihu sows was determined, and 23,921 probe sets were identified to have expression in the ovary follicles. Expression was detected for 16,066 transcripts (67.16% of all probe sets) in Large White ovarian follicles. A total of 15,477 transcripts (64.70% of all probe sets) were expressed in Chinese Taihu ovarian follicles (see additional file 1).

### Differentially expressed (DE) gene analysis

**Figure 1:**
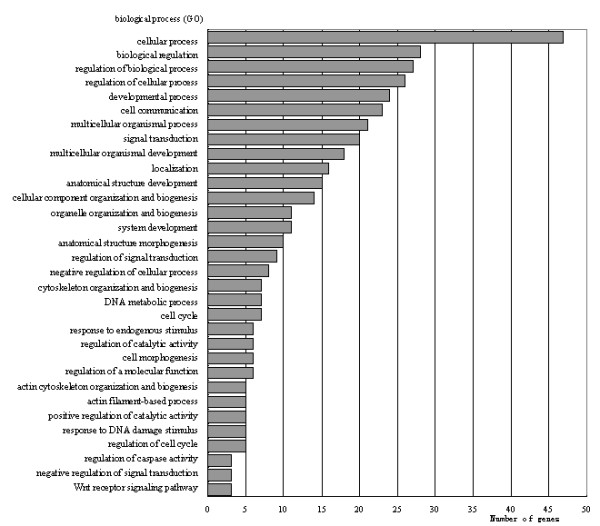
**Categories of 95 annotated transcripts in pigs or in human (FC ≥ 2 or ≤ 0.5, *p *≤ 0.05) based on biological process GO term**. Many categories shared the same transcripts.

The global expression profile of Chinese Taihu porcine ovaries challenged with PMSG-hCG was compared with that of the Large White sows. Probe sets whose intensities were normalized and filtered, then were subjected to identifying significantly differentially expressed genes using T-test analysis by comparing the log2 (normalized signal) of two breeds, and 1,476 transcripts were identified to be differentially expressed at the *p *≤ 0.05 level (see additional file 2). Fold change (FC) is the gene expression level (normalized signal intensity) of Chinese Taihu sows compared to Large White compared to Large White sows. Taking a FC ≥ 2 or ≤ 0.5 and the *p *≤ 0.05 significance level as the criteria, 133 transcripts showed differential expression. A set of 29 transcripts belonged to the up-regulated group and the other set of 104 transcripts belonged to the down-regulated group in Chinese Taihu sows (see additional file 3). Of the 133 DE transcripts, 12 were annotated pig genes, 83 transcripts could be determined for human homologous putative identities based on BLAST searches, and 38 additional probes did not have any database matches. Gene Ontology (GO) and Kyoto Encyclopedia of Genes and Genomes (KEGG) pathway analyses of 95 annotated DE gene lists were carried out by using the Database for Annotation, Visualization and Integrated Discovery (DAVID). GO annotation mapping revealed that the genes assigned GO terms for regulation of cellular process, regulation of biological process, biological regulation, developmental process, cell communication and signal transduction and so on (Figure 1). DE transcripts were involved in some important pathways including the WNT signaling pathway, p53 signaling pathway and Amyotrophic lateral sclerosis (ALS).

### Verification of gene expression pattern from microarray data by real-time RT-PCR

**Figure 2:**
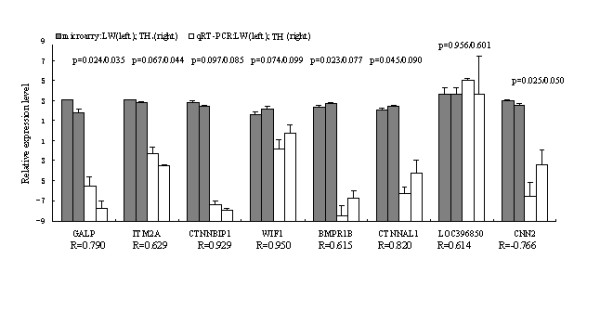
**Validation of the microarray data by quantitative real-time RT-PCR analysis of eight representative genes**. The x-axis represents the genes and the y-axis shows the relative expression (-ΔC_t _values for quantitative real-time RT-PCR; Log(Sample signal, 10) for microarray). The number of biological replicates is three for both assays. R represents the Pearson correlation coefficient. The significance of differences for the expression between Chinese Taihu (TH) and Large White (LW) PMSG-hCG stimulated ovary follicles was calculated using two-tailed T-test. *p *value: left for microarray, and right for quantitative real-time RT-PCR (qRT-PCR).

Eight genes (*GALP, ITM2A, CTNNBIP1, WIF1, BMPR1B, CTNNAL1, LOC396850*,*CNN2*) were selected to confirm the expression pattern by real-time RT-PCR. The results indicated that expression patterns of seven genes were consistent with the microarray (Pearson correlation coefficient ≥ 0.614, Figure 2). A contradiction expression profile was detected in *CNN2 *gene (Pearson correlation coefficient = - 0.766). This variance most likely came from technical differences.

### In *silico *mapping of DE genes to the interval of reproduction QTLs

Through BLAST of the 133 probes sets against Ensembl *Sus_scrofa *database (Sscrofa9, Apr 2009), 116 differentially expressed probes sets (transcripts) were *in silico *mapped pig chromosome covering 1-18 and X (see additional file 3). Ninety-two DE transcripts were located to porcine reproduction QTL chromosomal regions (see additional file 4). One such gene is the pig *BMPR1B *gene, which was localized to chromosome 8 within the interval of QTLs for ovulation rate, number of corpora lutea, litter size and prenatal survival [13-17]. Another gene is Kit-ligand/stem cell factor gene, which was located in the QTLs region for ovulation rate and FSH level [18,19].

### *BAX and BMPR1B *as the candidate genes for litter size

**Figure 3:**
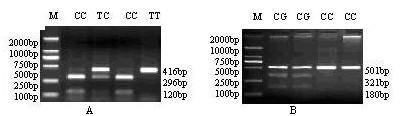
**Genotyping of single nucleotide polymorphisms in the porcine *BAX *and *BMPR1B *genes using PCR-RFLP**. (A): *BAX *gene, Genotype CC: 296 bp + 120 bp; Genotype TT: 416 bp; Genotype TC: 416 bp + 296 bp + 120 bp. (B): *BMPR1B *gene, Genotype GG: 321 bp + 180 bp; Genotype CC: 501 bp; Genotype CG: 501 bp + 321 bp + 180 bp.

*BAX *and *BMPR1B *were selected to be the candidate genes for litter size based on their biological functions on female reproduction and their positions within the intervals of QTLs. *BAX *T/C mutation in the intron 1 was genotyped as previously described [20], and *BMPR1B *C/G mutation was genotyped by PCR-*Sac*II-RFLP method which was established in the present study. As shown in Figure 3, the 416 bp *BAX *gene PCR amplicon containing T/C mutation was detectable by digestion with *Ear*I, resulting in allele T (416 bp) and allele C (296 and 120 bp) [20]; The 501 bp *BMPR1B *gene PCR amplicon containing C/G mutation was detectable by digestion with *Sac*II, resulting in allele C (501 bp) and allele G (321 and 180 bp). Genotyping results showed some variations in allele frequency between Chinese indigenous and Western breeds (Table 1). Briefly, the *BAX *allele C was fixed in Taihu, Tongcheng, Hezuo, Landrace, Duroc and White Duroc pigs; The *BMPR1B *allele C was fixed in Huainan, Tongcheng, Hezuo, Large White I, Landrace, Duroc, White Duroc and Pietrain pigs. Association analyses revealed that *BAX *CC had larger litter size than TC for both total number of piglets born (TNB) and the number of piglets born alive (NBA) of all parities in Large White pigs (*p *≤ 0.05), and for NBA of both the 1^st ^parity and all the parities in the DIV pigs (*p *≤ 0.05) (see additional file 5). The additive effects of *BAX *gene in Large White pigs ranged from 0.41 to 1.00 (*p *≤ 0.1). The *BMPR1B *CC was suggestively different with CG (*p *= 0.09) in NBA of all the parities in DIV line and the dominance effect was -0.66 piglet/litter (*p *= 0.06) (see additional file 5).

**Table 1:** Allele frequencies of *BAX and BMPR1B *gene polymorphisms in 11 pig populations

Populations	*BAX T/C*					*BMPR1B *C/G				
	
	No. tested pigs	No. TT	No. TC	No. CC	Allele T frequency	No. tested pigs	No. CC	No. CG	No. GG	Allele G frequency
Taihu	36	0	0	36	0.00	36	26	10	0	0.14
DIV	128	6	28	94	0.16	129	110	18	1	0.08
Huainan	26	0	7	19	0.14	26	26	0	0	0.00
Tongcheng	40	0	0	40	0.00	40	40	0	0	0.00
Hezuo	7	0	0	7	0.00	7	7	0	0	0.00
Large White I	108	4	38	66	0.21	108	108	0	0	0.00
Large White II	165	24	71	70	0.36	161	148	13	0	0.04
Landrace	16	0	0	16	0.00	13	13	0	0	0.00
Duroc	29	0	0	29	0.00	29	29	0	0	0.00
White Duroc	31	0	0	31	0.00	33	33	0	0	0.00
Pietrain	34	0	11	23	0.16	33	33	0	0	0.00

## Discussion

It is reported that a proportionately greater increase in ovulation rate occurs in Chinese Taihu after they have experienced pregnancy than with European breeds [21]. Using microarray expression profiling to identify changes in RNA levels resulting from genetic background, our studies revealed differential expression of 133 DE genes in ovarian follicle tissues at the preovulatory phase of the estrous cycle. Several genes not previously associated with the processes of follicle maturation or ovulation, were differentially expressed in follicle pools of the Large White and Chinese Taihu sows. Moreover, 83 probes with BLASTN matches to gene sequences of known function in human and 38 probes with no database matches, were also identified as being differentially expressed.

### Three DE gene pathways associated with p53 gene

WNT signaling pathway, p53 signaling pathway and Amyotrophic lateral sclerosis (ALS) were the major pathways including DE transcripts, and all of these three pathways are associated with p53 which was shown to promote apoptosis but not proliferation in ovarian granulosa cells [22,23].

The first evidence of the important role of WNT signaling in the ovary was provided by Vainio et al., which demonstrated that WNT4 mutant mice lost most of their oocyte reserve in the days prior to birth [24]. In the present study, six genes (*p53, WNT10B, TCF12, DKK2, CTNNBIP1, WIF1*) involved in WNT signaling pathway were differentially expressed in Large White and Chinese Taihu sows. DKK2 acts as an antagonist of WNT signaling to induce endocytosis of the WNT-Fz receptor complex [25]. *CTNNBIP1 *was found to negatively regulate WNT signaling via inhibition of the interaction between β-catenin and T cell factor (TCF) [26]. Wnt inhibitory factor 1 (WIF1), a secreted antagonist that can bind to WNT proteins directly and inhibit WNT signaling pathway, was also differentially expressed (fold change > 2, *p *= 0.07).

Pathway analysis found that two DE genes (*p53, BAX*) were involved in p53 signaling pathway and Amyotrophic lateral sclerosis. The BCL-2 associated X protein (BAX) is a member of the BCL-2 protein family and functions as an accelerator of apoptosis. The regulatory activity of BAX in follicular endowment likely occurs during primordial germ cell (PGC) migration, prior to PGC colonization of the gonad [27].

### Hormone related genes

Animal reproduction system is regulated by a suite of hormones. The serum hormone level of Chinese Taihu pigs was compared with the European in order to detect the mechanisms of high prolificacy of Chinese Taihu pigs. Chinese Taihu ovarian follicles tended to have a higher concentration of oestradiol in follicular fluid (*p *< 0.06) compared to Large White hybrids [28].

In our study, very limited hormone related genes were differentially expressed. One such gene encodes the caspase 8 associated protein 2 (CASP8AP2) which differentially suppresses steroid hormone receptor - induced transcriptional activity by interfering with their association with steroid hormone receptor coactivator 2 (SRC2)/N-CoA2 and SRC3/N-CoA3 [29]. Another one encodes cysteine - rich angiogenic inducer 61 (CYR61) which was known to be responsive to estrogen or to interact with estrogen receptor [30]. *GALP *gene encodes a member of the galanin family of neuropeptides and has been implicated in biological processes involving the central nervous system including hypothalamic regulation of metabolism and reproduction [31].

However, the well-known hormone related genes including estrogen receptor (*ESR*), follicle stimulating hormone (*FSH-β*) beta, retinol binding protein 4 (*RBP4*) genes had similar expressions in the two pig breeds. There are three major gene systems associated with follicular growth and ovulation: (1) *IGF-1*, estrogen and *FSH *gene, (2) the steroid receptors as well as orphan nuclear receptors and their ligands, and (3) the Wnt/Frizzled pathways [32]. In our study, their expressions of the genes from the first two systems probably were normalized by PMSG-hCG, and the genes from the last system remained differentially expressed.

### Positional candidate genes for female reproduction traits

Differentially expressed genes were *in silico *mapped to reproduction QTL regions. One of these genes is *BMPR1B *which played a pivotal role in bone formation during embryogenesis and fracture repair, and its mutation was associated with the increased ovulation rate of the Booroola sheep with FecB phenotype [33-35]. The pig *BMPR1B *gene was mapped to 108 cM on chromosome 8 [36], where the QTL for ovulation rate at approximately 105 cM, the QTL for number of corpora lutea at approximately 101 cM and 99 cM were reported to be located [13-15].

Another gene, the *KIT *gene encoding the transmembrane tyrosine kinase receptor KIT maps to the mouse W locus [37]. Kit-ligand/stem cell factor induces primordial follicle development and initiates folliculogenesis [38]. The pig *KIT *gene was located to 8p12-21, which is the same region as the QTL for ovulation rate at approximately 5 cM and for FSH level at approximately 12.4-11.1 cM [18,19,39].

### Two DE genes mutations and their associations with litter size

Marker-assisted selection (MAS) in conjunction with traditional selection methods is most effective for the traits such as litter size, which are either expressed later in life, are sex-dependent, or are of low heritability [40]. The candidate gene approach has led to notable success in demonstrating reproduction-related genetic markers or major genes, such as *ES*R, the erythopoietin receptor (*EPOR*), the prolactin receptor (*PRLR*), *FSH-β *and *RBP4 *[1,5,41-43]. Differentially expressed transcripts also can be evaluated as potential candidates if they meet the following criteria: the chromosomal location to a region containing a QTL, the importance of the tissue of interest in regulating the expression of the specific quantitative trait, and the critical developing stage responsible for the differences in the specific quantitative trait [44].

Here we selected the differentially expressed genes- *BAX *and *BMPR1B *as the candidate genes for litter traits due to their chromosomal location and/or their biological function. Some mutations were genotyped in several Chinese native pig breeds and Western pig breeds, and the results showed allele *BAX *C and *BMPR1B *C were fixed in several tested pig populations including Huainan, Tongcheng, Landrace, Duroc, White Duroc and Pietrain pigs. We also noticed that in DIV line and Large White pigs allele *BAX *C and *BMPR1B *C were not fixed, but *BAX *TT and *BMPR1B *GG did not nearly appear, possibly because the homozygotes led to embryonic lethality in these specific pig populations. The association analyses of these mutations with litter traits were carried out in Large White and Chinese DIV line pigs. The *BAX *gene was significantly associated with the litter size in these two populations, suggesting this mutation might be a good marker for pig selection and breeding. Suggestive association (*p *= 0.09) was found between the *BMPR1B *and litter size, in some ways similar to the results of Tomas et al., which also only found suggestive associations (nominal *p *< 0.1) between the *BMPR1B *haplotypes and the number of piglets born alive and weaned when data from the first parity of Iberian × Meishan F2 sows were considered [45].

## Conclusions

We have reported differential gene expression in ovarian follicles at the preovulatory stage of a PMSG-hCG stimulated estrous cycle of two pig breeds with different ovulation rate. Some differentially expressed genes and key pathways related to biological and cellular regulation were identified. Furthermore, two differentially expressed genes- *BAX *and *BMPR1B *genes were genotyped and associated with the litter size traits in Large White and Chinese DIV pigs. The results of this study provide an opportunity to elucidate the genetic control of ovulation rate and improve our understanding of high prolificacy of Chinese Taihu pigs.

## Methods

### Population and experimental design, tissue collection

All animal procedures were performed according to protocols approved by the Biological Studies Animal Care and Use Committee of Hubei Province, PR China. Three multiparous Taihu cyclic sows (TH; ≥ 2 parities) from Jiangsu Changshu Xuqin Corporation and 3 multiparous Large White cyclic sows (LW; ≥ 2 parities) from Jinpin farm of Huazhong Agricultural University that exhibited normal estrous cycles were treated with 1000 IU PMSG and 500 IU hCG as previously described [46]. Given that the difference of prolificacy between Large White and Chinese Taihu pigs possibly due to the differences in the characteristics of follicle maturation [28], PMSG-hCG was used to initiate and synchronize the follicular phase. PMSG-hCG stimulated sows were killed and the ovaries at the preovulatory phase were removed immediately and kept in ice-cold physiological saline (0.9% wt/vol NaCl) during dissection of follicles. Only healthy follicles with a diameter of > 5 mm were isolated and snap-frozen in liquid nitrogen [2]. Total RNA was isolated from ovary follicles with Trizol (Invitrogen). RNA yield was quantified by spectrophotometric analysis using the convention that 1 absorbance unit at 260 nm equals 40 μg/mL RNA.

Animals from 11 different populations were used to investigate the allele frequency, including 36 Chinese Taihu pigs, 40 Tongcheng pigs, 7 Hezuo pigs, 26 Huainan pigs, 129 pigs from DIV line (the 4^th ^Dam line of Chinese lean-type new lines), 108 Large White pigs I, 165 Large White pigs II, 16 Landrace pigs, 29 Duroc, 33 White Duroc and 34 Pietrain pigs.

The association analyses were conducted in Large White and DIV pig populations. Two hundred and seventy three Large White pigs contained 108, 165 pigs from strain I and II, which were raised in the farms owned by Huazhong Agricultural University (HAU) and Hubei Institute of Animal Science and Veterinary Medicine, respectively. Synthetic Line DIV was a result of cross of Landrace, Large White, Tongcheng or Taihu pigs, and was raised in pig farm owned by HAU. During the consecutive years (2005-2010), TNB and NBA of animals were recorded in 491 litters of DIV Line sows and in 481 litters of Large White II sows.

### Microarray hybridizations and data analysis

The RNA labeling and hybridization were conducted by a commercial Affymetrix array service according to Technical Manual of GeneChip^® ^Expression Analysis (CapitalBio Corporation, Beijing, China). Briefly, a total of 5 μg RNA was converted to double - stranded cDNA using the one-cycle cDNA Synthesis Kit (Affymetrix, Inc., Santa Clara, CA) and T7-Oligo (dT) Primer. In vitro transcription (IVT) of cRNA from cDNA was conducted using the MEGAscript ^® ^T7 Kit (Ambion, Inc.). After the cleanup of the cDNA and cRNA using the Sample Cleanup Module (Affymetrix), the GeneChip IVT Labeling Kit (Affymetrix) was used for synthesis of Biotin-Labeled cRNA. cRNA quality and concentration was checked by UV spectrophotometer analyses and 2 μg cRNA was then checked by formaldehyde denaturing gel electrophoresis in 1.2% agarose gel. Subsequently, labeled cRNA was fractionated and hybridized with the GeneChip Porcine Genome Array according to the standard procedures provided by the manufacturer. Chips were washed and stained with a GeneChip Fluidics Station 450 (Affymetrix) using the standard fluidics protocol. The probe arrays were scanned using the Affymetrix ^® ^GeneChip^® ^Scanner 3000. Six microarrays were used in the experiment, corresponding to the RNAs from PMSG-hCG stimulated preovulatory ovarian follicles of three Taihu sows and three Large White sows. The Affymetrix GeneChip Porcine Genome Array probe set contained 11 pairs of perfect match (PM) and mismatch (MM) 25-mer probes. The probe-pair (PM-MM) data were used to detect the expression level of genes on the array (present call, marginal call, and absent call) by MAS 5.0 (Wilcoxon signed rank test). MAS 5.0 was used to normalize the signal values above to 500 on each array. An invariant set normalization procedure was performed to normalize the different arrays using DNA-chip analyzer (dChip). Probe sets whose intensity were above 50 on at least three arrays were remained and then subjected to identifying significantly differentially expressed genes. Identification of DE genes in Large White and Chinese Taihu was conducted using SAS (Ver.8.1, T-test) by comparing the log2 (normalized signal) of two breeds. The p value cutoff for DE genes was set at 0.05. The data discussed in this publication have been deposited in NCBI's Gene Expression Omnibus and are accessible through GEO Series accession number GSE23985 http://www.ncbi.nlm.nih.gov/geo/query/acc.cgi?acc=GSE23985. Genes with significant similarities to the transcripts in nr database based on BLAST, were selected for GO analyses (EASE threshold = 0.1, count threshold = 2) and pathway analysis at the DAVID http://david.abcc.ncifcrf.gov/. Annotation summary results were obtained by inputting the gene list of interest by selecting OFFICIAL_GENE_SYMBOL as identifier.

### Real-time RT-PCR to analyze gene expression profiles

Total RNAs were treated with DNase I (Ambion) and reverse transcribed by the M-MLV Reverse Transcriptase (Promega) according to the manufacturer's instructions. Quantitative real time PCR was performed on the iQ™5 Real Time PCR Detection System (Bio-Rad) using SYBR^® ^Green Real-time PCR Master Mix (Toyobo Co., Ltd., Japan). Each 25 μL real-time RT-PCR reaction included 12.5 μL SYBR Green Real-time PCR Master Mix, 350-500 ng cDNA, 0.3 μM primers (see additional file 6). PCR conditions consisted of 1 cycle at 95°C for 3 min, followed by 45 cycles at 94°C for 20 sec, 58°C for 20 sec, and 72°C for 18 sec, with fluorescence acquisition at 74°C. cDNAs from three Taihu and three Large White ovary follicles were used as the template to detect the expression changes of the target genes. All PCRs were performed in triplicate and gene expression levels were quantified relatively to the expression of β-actin using Gene Expression Macro software (Bio-Rad, Richmond, CA, USA) by employing Δ*C*_t _value. Student's *t*-test was conducted to identify differentially expressed genes. Due to the negative relationship between C_t _and expression level, an improved method of the previous report was used to compare the results of real time RT-PCR and microarray by plotting the -Δ*C*_t _values of real time RT-PCR versus the log of the microarray signal for each gene [47]. The Pearson correlation coefficient was calculated and used to estimate the correlation of real time RT-PCR results and microarray results.

### Integration of differentially expressed genes in porcine QTL regions

To retrieve the corresponding pig gene data of one differentially expressed EST, the probe (EST) sequence was used as a query to search homology using BLASTN against Ensembl *Sus_scrofa *database (Sscrofa9, Apr 2009). QTLs for reproduction traits were obtained from the PigQTLdb http://www.animalgenome.org/cgi-bin/QTLdb/SS/browse. The differentially expressed probe sets located in these QTL intervals were identified through comparison analysis [11]. The reproduction traits included TNB, NBA, fully formed piglets, number of stillborn, ovulation rate, age at puberty and uterine capacity. We listed all Affymetrix probe sets that were located in these QTL intervals.

### Detection of SNPs of differentially expressed genes and association analyses

Two differentially expressed genes- *BAX *and *BMPR1B *were selected as potential candidates for litter size due to their functions and/or chromosomal positions, and further were investigated for their effects on litter traits. PCR-RFLPs were used to identify their mutations including a C/T at intron 1 of *BAX *gene [20], and a C/G of *BMPR1B *gene (Fan B, private communication). Briefly, PCR reactions contained 1.5 mM MgCl_2_, 150 μM dNTPs, 0.5 μM of each PCR primers (see additional file 6), 1 μL DNA, and 1 U *Taq *DNA polymerase in a 25 μL volume. PCR was run as follows: 94°C for 4 min followed by 35 cycles of 94°C for 40 sec, 60°C for 40 sec, 72°C for 35 sec, and final extension of 72°C for 10 min. For PCR-RFLP profile, 8.5 μL of PCR products were digested with 5 U restriction endonuclease (TaKaRa) for 4 h at 37°C, and then separated by electrophoresis on a 1-3% agarose gel (with ethidium bromide) in 1 × TAE buffer. The relationships between differentially expressed genes genotypes and litter size traits of Large White (n = 273) and DIV (n = 129) pigs were evaluated with the general linear model (GLM) procedure of SAS version 8.0. The model: *Y*_*ijkl *_= *μ *+ *P*_*i *_+ *S*_*j *_+ F_*k*_+ *G*_*l *_+ *e*_*ijkl*_, where *Y*_*ijkl *_is the observation of the trait, *μ *is the least square means, *P*_*i *_is the effect of *i*th parity (*i *= 1, 2, 3, 4 (parity ≥ 4 )), *S*_*j *_is the effect of *j*th season, *F*_*k *_is the effect of *k*th farm (*k = 1, 2*), *G*_*l *_is the effect of *l*th genotype (*l *= *1-3*) and *e*_*ijkl *_is the random residual [48].

## Abbreviations

DE: differentially expressed; FC: fold change; GO: gene ontology; QTL: quantitative trait loci; Chinese DIV line: the 4th dam line of Chinese lean-type new line; GALP: galanin-like peptide; ITM2A: integral membrane protein; CTNNBIP1: catenin, beta interacting protein 1; WIF1: Wnt inhibitory factor 1; BMPR1B: bone morphogenetic protein receptor, type IB; CTNNAL1: catenin (cadherin-associated protein) alpha-like1; CNN2: calponin 2; P53: p53 protein; BAX: BCL-2 associated X protein; kit: v-kit Hardy-Zuckerman 4 feline sarcoma viral oncogene homolog; ESR: estrogen receptor; PRLR: prolactin receptor; FSH-β: follicle-stimulating hormone β; EPOR: erythopoietin receptor; RBP4: retinol binding protein 4; TCF: T cell factor; MAS: marker-assisted selection; KEGG: Kyoto Encyclopedia of Genes and Genomes; TNB: total number of piglets born; NBA: the number of piglets born alive.

## Authors' contributions

XS carried out most of bioinformatics analysis and lab works. SM participated in the animal samples collection and statistical analysis. HT, GW, LS made substantial contributions to lab works. SJ, CD, YX participated in the experiment design and coordination. FL conceived the study and drafted the manuscript. All authors read and approved the final manuscript.

## Supplementary Material

Additional file 1 **Ovarian follicle transcriptome analyses using the Affymetrix Porcine Genechip**. Data of each probe is from the three Large White sows (1, 2, 3) and three Chinese Taihu (1, 2, 3) sows. Raw data from. CEL files were converted to gene signal files and raw signals were normalized above to 500 on each array by MAS 5.0, and the dChip invariant set was used to normalize the signals of the different arrays. "P", present; "A", absent; "M", marginal; "Count (P)", the number of P flag; select while Count (P) ≥2 in two groups (Chen et al., 2009)*. Totally, 16,066 and 15,477 probesets were detected expression in the Large White and Taihu, respectively. *Chen H, Li C, Fang M, Zhu M, Li X, Zhou R, Li K, Zhao S. Understanding Haemophilus parasuis infection in porcine spleen through a transcriptomics approach. BMC Genomics. 2009, 5, 10:64. Click here for file

Additional file 2 **1476 transcripts that are differentially expressed in porcine ovary between Large White sows and Chinese Taihu sows (p ≤ 0.05)**. Totally 105 (row 5-109) transcripts have been annotated based on BLASTX searches. "FC", Fold change, gene expression level (normalized signal intensity) of Chinese Taihu sows compared to Large White sows, ">1" represents up regulation, "<1" represents down regulation. "p-value", significance level of differential expression for a particular gene by comparing their log 2 (normalized signal). "Gene name", top informative BLASTX hit. Click here for file

Additional file 3 **Pig unique genes (row 4-15), unique genes with BLSATX identity but no annotation (row 16-98) and unique genes with no BLASTX identity (row 99-136) (p ≤ 0.05, FC ≥ 2 or ≤ 0.5)**. "FC", Fold change, gene expression level (normalized signal intensity) of Chinese Taihu sows compared to Large White sows, "≥2" represents up regulation, "≤0.5" represents down regulation. "*p*-value", significance level of differential expression for a particular gene by comparing their log 2 (normalized signal). "Gene name", top informative BLASTX hit. Click here for file

Additional file 4 **Genes that distributed in the intervals of the known pig QTLs for reproduction traits**. All the genes listed here are from 133 transcripts that are significantly up/down-regulated between Large White and Taihu sows. GenBank IDs represent annotated genes. Click here for file

Additional file 5 **Association between *BAX *and *BMPR1B *genotype and litter size traits**. ^A ^and ^B^, *p *≤ 0.05; ^a ^and ^b^, *p *= 0.09. ** *p *≤ 0.05, *p ≤ 0.1 = 0.06. N: Number of investigated litters. Click here for file

Additional file 6 **Primer pairs designed for genes selected for validation by real time RT-PCR and SNP detection**. Click here for file
