# Supplementary material for: Microarray profiling for differential gene expression in PMSG-hCG stimulated preovulatory ovarian follicles of Chinese Taihu and Large White sows
Source: BMC Genomics. 2011 Feb 16;12:111. doi: 10.1186/1471-2164-12-111 (PMC3047302; doi:10.1186/1471-2164-12-111)
Supplement: Additional file 5 — Association between BAX and BMPR1B genotype and litter size traits. A and B, p ≤ 0.05; a and b, p = 0.09. ** p ≤ 0.05, *p ≤ 0.1 = 0.06. N: Number of investigated litters. [file 1471-2164-12-111-S5.DOC]

**Additional file 5: Association between *BAX* and *BMPR1B* genotype and litter size traits**

| Popul-  ations | parity | Traits | *BAX* Genotype (μ ± SE) | | | Effect (μ ± SE) | |
| --- | --- | --- | --- | --- | --- | --- | --- |
|  |  |  | TT | TC | CC | Additive | Dominance |
| Large White  pigs | 1^st^ parity | N | 12 | 50 | 62 |  |  |
|  |  | NBA | 8.00±0.82 ^A^ | 8.48±0.40^A^ | 9.83±0.37 ^B^ | 0.92±0.45^**^ | -0.22±0.30 |
|  |  | TNB | 8.66±0.82 ^A^ | 9.61±0.41^A^ | 10.68±0.37 ^B^ | 1.00±0.31^**^ | -0.03±0.30 |
|  | all parities | N | 37 | 178 | 260 |  |  |
|  |  | NBA | 8.30±0.79 ^A^ | 8.72±0.29 ^A^ | 9.93±0.26 ^B^ | 0.51±0.25^**^ | -0.17±0.16 |
|  |  | TNB | 9.62±0.78 | 10.11±0.29^a^ | 10.76±0.26^b^ | 0.41±0.23^*^ | -0.04±0.15 |
| Line  DIV  pigs | 1^st^ parity | N | 2 | 15 | 45 |  |  |
|  |  | NBA | 6.50±1.86 | 8.67±0.68 ^A^ | 9.36±0.69 ^B^ | 1.43±0.95 | 0.37±0.58 |
|  |  | TNB | 11.00±1.73 | 9.67±0.63 | 11.11±0.37 | 0.06±0.88 | -0.69±0.54 |
|  | all parities | N | 31 | 120 | 340 |  |  |
|  |  | NBA | 9.83±0.65 | 9.86±0.32^A^ | 10.37±0.18^B^ | 0.18±0.27 | -0.27±0.19 |
|  |  | TNB | 11.17±0.52 | 10.58±0.26 | 11.22±0.16 | 0.10±0.27 | -0.27±0.19 |
| Popul-  ations | parity | Traits | *BMPR1B* Genotype (μ ± SE) | | | Effect (μ ± SE) | |
|  |  |  | CC | CG | GG | Additive | Dominance |
| Large White  pigs | 1^st^ parity | N | 117 | 10 | 0 |  |  |
|  |  | NBA | 9.07±0.27 | 9.20±0.91 | 0 | 0.13±0.96 | 0 |
|  |  | TNB | 10.00±0.28 | 10.10±0.93 | 0 | 0.09±0.97 | 0 |
|  | all parities | N | 445 | 36 | 0 |  |  |
|  |  | NBA | 9.44±0.14 | 9.62±0.47 | 0 | 0.24±0.50 | 0 |
|  |  | TNB | 10.42±0.13 | 10.67±0.45 | 0 | 0.30±0.47 | 0 |
| Line  DIV  pigs | 1^st^ parity | N | 53 | 8 | 1 |  |  |
|  |  | NBA | 9.06±0.37 | 9.50±0.95 | 8.00±2.68 | -0.53±1.35 | 0.46±0.82 |
|  |  | TNB | 10.89±0.34 | 10.00±0.89 | 10.00±2.51 | -0.44±1.27 | -0.22±0.77 |
|  | all parities | N | 388 | 98 | 5 |  |  |
|  |  | NBA | 10.34±0.15 | 10.01±0.29 | 12.04±1.28 | 0.84±0.65 | -0.56±0.35 |
|  |  | TNB | 11.15±0.15^a^ | 10.60±0.29^b^ | 12.60±1.28 | 0.72±0.64 | -0.66±0.35^*^ |

Note: ^A^ and ^B^, *p ≤* 0.05; ^a^ and ^b^, *p* = 0.09. ^**^ *p ≤* 0.05, **p ≤* 0.1= 0.06. N: Number of investigated litters.
